# Patient interviews in interprofessional and intercultural contexts (PinKo) – project report on interdisciplinary competence development in students of medicine, pharmacy, and community interpreting

**DOI:** 10.3205/zma001463

**Published:** 2021-03-15

**Authors:** Kai-Uwe R. Strelow, Şebnem Bahadır, Bettina Stollhof, Rita M. Heeb, Holger Buggenhagen

**Affiliations:** 1 University of Mainz, University Medical Center, Rudolf Frey Lernklinik, Mainz, Germany; 2 University of Mainz, Translation, Linguistics and Cultural Studies, Department of Intercultural German Studies, Germersheim, Germany; 3 University of Graz, Department of Translation Studies, Graz, Austria; 4 University of Mainz, Institute of Pharmacy and Biochemistry, Clinical Pharmacy, Training Pharmacy, Mainz, Germany

**Keywords:** interdisciplinary teaching, interprofessional courses, intercultural competence, culture, migration, interpreters, patient interviews, treatment safety, patient safety

## Abstract

**Background: **Hospitals and other medical institutions must prepare for a further increase in patients who are either immigrants or Germans with a migration background. In spite of the unquestionable educational and socio-political relevance of this topic, most German universities do not offer a comprehensive curriculum aimed at increasing intercultural awareness and putting it into practice in the training of students in medicine and pharmacy. Against this background, this article presents the innovative teaching project “Die Triade”, which was jointly implemented by the Departments of Medicine, Pharmacy and Translation Studies at the University of Mainz.

**Aim:** The aim is to give an overview of the development, realisation, implementation and consolidation of the course “Patient interviews in interprofessional and intercultural contexts” (PinKo), which was designed in the project “Die Triade”.

**Project description: **A two-day course was developed, starting with a block session for all participating students to teach the basics of interprofessional and intercultural competence development. On the second practical training day, students learn and practice triadic conversation in different language groups using scripted roles. While the trainee doctors and pharmacists represent their respective professions in the prepared conversational situations, the interpreting students take on the roles of interpreters and patients. The event is jointly supervised by lecturers from the participating professions and language groups.

**Results:** In the 2016 summer semester and the following winter semester, the course was organised for a total of 112 students. The event as a whole was evaluated by means of a questionnaire by the students of the participating departments (Medicine (M) N=8, Pharmacy (P) N=60; Translation (T) N=44). Overall, the event was rated as good (1=very good, 6=insufficient) ((M) 1.67/2.00; (P) 2.29/3.33; (T) 1.50/1.86). The course tended to be rated lower by pharmacy students; this also applies to the rating of the development of interprofessional competences ((M) 1.33/2.00, (P) 2.00/2.93, (T) 1.82/2.25).

**Discussion: **The course is suitable for the acquisition of interprofessional as well as intercultural competences. However, in order to improve the course in a participant-centred way, train larger numbers of participants and include additional healthcare occupations such as nursing or assistant medical professions, adaptations of the concept would be necessary. In this context, the digitalisation of the learning content appears to be particularly useful for ensuring that the course can be adapted to heterogeneous groups of participants and to optimise in-person times for further opportunities for practice.

## Zusammenfassung

**Hintergrund: **Krankenhäuser und andere medizinische Einrichtungen müssen sich auf eine weitere Zunahme von Zugewanderten und Deutschen mit Migrationshintergrund als Patienten einstellen. Trotz der außer Frage stehenden bildungs- und gesellschaftspolitischen Relevanz gibt es an den meisten deutschen Universitäten jedoch kein übergreifendes curriculares Angebot in der Ausbildung von Medizin- und Pharmaziestudierenden für eine interkulturelle Sensibilisierung und deren Umsetzung in praktisches Handeln. Vor diesem Hintergrund stellt dieser Beitrag das innovative Lehrprojekt „Die Triade“ vor, das gemeinsam von den Fachbereichen Medizin, Pharmazie und Translationswissenschaft der Universität Mainz realisiert wurde.

**Ziel: **Das Ziel ist es, einen Überblick über die Entwicklung, Durchführung, Implementierung und Verstetigung der Lehrveranstaltung Patientengespräche im interprofessionellen und interkulturellen Kontext (PinKo) zu geben, die im Rahmen des Projekts „Die Triade“ konzipiert wurde.

**Projektbeschreibung: **Entwickelt wurde eine zweitägige Veranstaltung, beginnend mit einer Blockveranstaltung für alle teilnehmenden Studierenden zur Vermittlung von Grundlagen zur interprofessionellen und interkulturellen Kompetenzentwicklung. Am zweiten sogenannten Praxistag erlernen und üben die Studierenden anhand von vorbereiteten Rollenskripten die triadische Gesprächsführung in verschiedenen Sprachengruppen. Während die angehenden Ärzt*innen und Pharmazeut*innen ihre Professionen in den vorbereiteten Gesprächssituationen vertreten, übernehmen die Dolmetschstudierenden die Dolmetscher- und Patientenrollen. Die Veranstaltung wird gemeinschaftlich von Dozierenden der beteiligten Professionen und Sprachengruppen begleitet.

**Ergebnisse:** Im Sommersemester 2016 und dem folgenden Wintersemester wurde die Veranstaltung für insgesamt 112 Studierende durchgeführt. Dabei wurde die Gesamtveranstaltung mittels Fragebogen von den Studierenden der beteiligten Fachbereiche (Medizin (M) n=8, Pharmazie (P) N=60; Translation (T) N=44) bewertet. Insgesamt wurde die Veranstaltung mit gut (1=sehr gut, 6=ungenügend) beurteilt ((M) 1.67/2.00; (P) 2.29/3.33; (T) 1.50/1.86). Die Veranstaltung wurde von den Pharmaziestudierenden tendenziell schlechter bewertet, was auch für die Bewertung der interprofessionellen Kompetenzentwicklung ((M) 1.33/2.00, (P) 2.00/2.93, (T) 1.82/2.25) gilt.

**Diskussion: **Die Lehrveranstaltung ist als Lehrangebot sowohl für den Erwerb interprofessioneller als auch interkultureller Kompetenzen geeignet. Um die Veranstaltung teilnehmerzentriert zu verbessern, größere Teilnehmerzahlen zu schulen und weitere Gesundheitsberufe wie die Pflege oder medizinische Assistenzberufe miteinzubeziehen, wären allerdings Anpassungen des Konzepts notwendig. Hierbei erscheint insbesondere die Digitalisierung der Lerninhalte sinnvoll, um so eine Anpassung der Veranstaltung an heterogene Teilnehmergruppen zu gewährleisten sowie die Präsenzzeit für weitere Übungsmöglichkeiten zu optimieren.

## 1. Introduction

According to the 2018 migration report of Germany’s Federal Office for Migration and Refugees (BAMF), approximately one in four people living in Germany (25.5%) has a migration background [1]. Hospitals and other medical institutions must be prepared for a further increase in patients with little or no knowledge of German [1], [2]. Due to this development, hospitals and medical practices in Germany are confronted with an increase in interculturality, multilingualism and a higher effort for communication. Although this development is not new, it has gained momentum in recent years, especially due to the increasing number of migrants and people in need of protection. This is accompanied by the corresponding challenges for the healthcare system in securing the health policy goal of ensuring equal care for all patient groups [2], [3], [4]. Despite the unquestionable educational and socio-political relevance, German universities do not offer a comprehensive curriculum in the education of medical and pharmacy students for increasing intercultural awareness and putting it into practice. Courses in pilot degree programmes like those at the University Medical Center Mannheim (pilot degree programme “MaReCuM”) or at the medical faculties in Hamburg (pilot degree programme “iMED”) or the Charité in Berlin (New Revised Medical Curriculum) have so far remained an exception [5].

In order to achieve a shared diagnostic and therapeutic understanding, there is an increased need for counselling in medical care in Germany, taking into account linguistic and cultural difficulties. In addition to sociographic and medical factors such as age, gender, social and familial situation as well as the health status of the patients, migrants often also experience language problems in medical consultation and care [2], [3], [4], [6]. In this context, problems involving language are often regarded as a cause of dissatisfaction for everyone involved in the conversation, which can either prevent or unnecessarily reinforce the perception of cultural differences that are either not understood or misunderstood. They can also contribute to misunderstandings and errors in medical-pharmaceutical advice and thus have a detrimental effect on safe patient care. In this regard, culturally sensitive communication is of particular importance. There are by now a considerable number of reports, recommendations for action and guides highlighting that measures to improve intercultural competences bring major benefits to counselling and treatment in applied medical practice [7], [8]. Among other things, these include

- optimisation of the interaction with patients and relatives,
- greater satisfaction of all parties involved when dealing with intercultural situations,
- avoidance of human error,
- more patient-oriented consultation and treatment, and
- an increase in treatment quality and safety.

Since the opportunities for intercultural qualification in the healthcare professions are still far from adequate in Germany, the development of course formats for training and continuing education plays an important role in promoting intercultural competence in everyday medical practice and in establishing culturally sensitive healthcare.

This paper presents the initial results of the development and implementation of an innovative and interprofessional course in the degree programmes of medicine, pharmacy and translation studies. The course was designed jointly by the faculties of Medicine, Pharmacy, and Translation, Linguistics and Cultural Studies (FTSK) of the Johannes Gutenberg University in Mainz (JGU) in the context of an innovative teaching project (“Die Triade”) that was funded by the university [9].

## 2. Teaching in an interprofessional and intercultural context

### 2.1. Interprofessional teaching

The idea of bringing professions that cooperate closely in a professional medical field together in education, training and continuing education as well was already addressed in the 1960s and taken up in subsequent years by the World Health Organization (WHO) [10]. Since then, numerous studies and meta-analyses on interprofessionally oriented teaching have been initiated under the banner of Interpersonal Education (IPE). IPE concepts are defined as “... shared learning experiences among health profession students across disciplines, with the goals of professional identification, the edification of strong clinical teams and the improvement of health outcomes” [11] (p.144).

Central to this is the goal of cultivating the collaborative practice of patient-centred healthcare through shared and part-time training components in order to, in a manner or speaking, offset deficiencies in collaboration [12]. In addition to the medical profession and nursing, pharmacy and other healthcare occupations are also mentioned. These last only play a minor role, as do social professions involved in healthcare [11], [13], [14]. With regard to the impact of IPE, the studies generally mention improvements in the areas of inter-professional communication and cooperation [11], [12], [13], [15]. Joint training also leads to a deeper understanding of responsibilities and clear roles in the shared professional field.

As the use of interprofessional training reduces deficits in the area of collaboration, there are improved outcomes in providing patients with information, as well as a decrease in errors. This results in a correlation between the use of IPE and successful treatment, patient safety, and an increase in the overall competence of the team [11], [12], [16], [17]. In addition to the knowledge and the previously mentioned effects in the areas of communication and teamwork, the development of common fundamental ethical attitudes and approaches [10], [12], as well as the strengthening of trust and respect [15] are mentioned here.

These findings support the call for mandatory interprofessional training in the education of healthcare professionals [18]. However, there are numerous obstacles to this. In addition to insufficient funding and the often inadequate geographical proximity of the professions involved, the lack of motivation on the part of the faculties and deficits in institutional management are mentioned here, along with problems aligning curricula and awarding course credits and differences in pedagogical approaches [11]. In this respect, beyond the willingness of the professions involved, it is necessary for the university to create a programmatic and interprofessionally oriented infrastructure in order to successfully establish IPE as a promising key factor in education [14], [15], [19].

In its 2014 report, the German Council of Science and Humanities (WR) also criticized the lack of scope for didactic innovation in medicine. At the same time, in connection with the reform and competence orientation of medical training in Germany, it calls for more interprofessional cooperation in teaching and practice [5]. In 2017, the Healthcare/Medical round table and health scientists of the project “nexus – forming transitions, promoting student success” that was conducted by the German Rectors’ Conference (HRK) also focused on interprofessional teaching and learning at universities for medicine and the qualified healthcare professions [20]. 

The teaching project presented here goes one step further: interprofessional cooperation does not only involve the health, nursing and social professions (as the recently published nursing report for 2019 emphasizes several times, cf. for this especially the contribution by Behrend et al. 2020) [21] – it also includes the interpreting profession. This expansion of interprofessional training with the vision of improved culturally sensitive medical practice is unique in the German-speaking world. On an international level, only the research project “EmpathicCare4All” at the universities of Leuven and Ghent in Belgium can be mentioned here; it develops comparable courses according to IPE for medical and interpreting students [22].

#### 2.2. Intercultural competence in the healthcare professions

There is widespread agreement in the research community that promoting intercultural competence is the most crucial measure to address the previously mentioned challenges in healthcare [23], [24], [25], [26]. Based on a processual model that does not regard intercultural competence as a separate area of competence but as a context-related variant of general agency, it can be understood as a “synergetic” product of existing partial competences, i.e. of professional, methodological and social or interpersonal competence [27]. In contrast to the professional profile of community interpreters [28], [29], [30], intercultural competence is not a key qualification for doctors and pharmacists, even if the students are not unfamiliar with the subject matter due to the (communicative) agency they have already acquired. However, the development of intercultural competences requires a stronger focus on the counselling and treatment process of individuals who do not speak German. Studies and health policy reports such as the focus report on migration and health (2008) issued by Germany’s federal health monitoring show that migrants behave differently than locals who speak the language and know the healthcare system, especially when it comes to accessing healthcare and making initial contact. In this context, the report highlights the role and availability of training for healthcare professionals, interpretation services and multilingual information resources [31], [32]. That perceptions of illness, health, suffering and pain, as well as ideas regarding the role of medical personnel and therapeutic measures can vary according to culture is not a new finding for research in medical ethnology, medical anthropology and transcultural medicine. In the context of migration, this gains new significance for culturally sensitive standard care in Germany. For healthcare to be sensitive to differences, measures are needed that reduce barriers to access for patient groups with different cultural backgrounds and give greater consideration to cultural diversity and multilingualism in medical consultations and treatment. It is often assumed that a non-verbal articulation of illness or illness-specific suffering is easier to understand and can serve as a basis for communication. However, healthcare professionals tend to be insufficiently aware of, view as exaggerated, or trivialise expressions of pain and suffering from different cultures. Empathy and genuine interest as well as access to and understanding of culture-specific forms of articulation modes, conceptualisations and coping strategies are therefore cornerstones of transcultural competence [4], [32]. The ability of medical staff to recognise and reflect on their own ethnocentric or culturalising approaches is bolstered significantly by the involvement of community interpreters. This insight plays a central role in the teaching project “Die Triade” [28], [30].

#### 2.3. Use of language mediators in the healthcare sector

So far, there are only estimates that roughly one in three patients with a migration background has a need for professional interpreting services [4]. In this context, the recruitment of staff with a migration background and the use of interpreters becomes particularly controversial. In the field of translation studies, the importance of community interpreters specifically trained for this field of work, as well as the potentials and risks of using family members, untrained healthcare personnel and other laypersons as language mediators, has been researched in recent years [28], [33], [34], [35]. Studies with non-professional interpreters show that interpreting is often incorrect or incomplete due to a lack of specialised knowledge, but also because the interpreter is emotionally impacted. Trained community interpreters, on the other hand, act responsibly and in accordance with a professional code of ethics that focuses on multi-partiality. In addition to the social interaction between the medical staff and the patient, they also consider the socio-cultural aspects of illness and medical-therapeutic measures relevant to the individual case. While patients have an obligation to point out communication problems [36], the doctor must ensure that the patient, in consenting, has understood the essential information regarding their illness, the treatment options and the associated risks. This is particularly important in the case of informed-consent interviews. According to a ruling by Germany’s Federal Social Court in 2006, patients are not entitled to treatment or consultation in their native language [37]. However, Germany’s 2013 Patients’ Rights Act stipulates that medical information must also be comprehensible for patients with little or no knowledge of German. According to a study on the effects of the Patients’ Rights Act, however, this vulnerable group of patients is largely left to their own devices, as professional interpreters are often not used and interpreting costs are not covered [38]. In practice, a variety of compensatory measures are used to offset language deficits. Thus, in addition to using another language, such as English, there is often an attempt to speak more slowly or loudly, to formulate what is said more simply and to emphasise it more. However, strategies of this type are sometimes perceived as inappropriate or degrading and can lead to counter-reactions and behaviours in patients that are not conducive to obtaining informed consent [4]. Other possibilities for facilitating communication can be found in the non-linguistic and visual areas. In addition to indicating and touching parts of the body relevant to examination or treatment, illustrations and visualized diagnostic results also appear to be helpful. To a large extent, this also applies to patient information sheets that are available in other languages and include visual explanations. Whether a community interpreter is required is therefore a case-by-case decision that, according to the current Patients’ Rights Act, is the responsibility of the physician who is providing treatment. However, this requires healthcare professionals to be made aware of cultural differences and how to work with interpreters.

## 3. Project description and methodology

### 3.1. General conditions

For the interdisciplinary teaching project, culturally sensitive interprofessional cooperation and the development of intercultural competences were already established as essential cornerstones for the development and implementation of the course when the application was submitted. Despite the different training conditions of the participating disciplines, it was possible to identify courses and formats in which the PinKo training could be integrated. In translation studies, for example, PinKo is offered as part of the Community Interpreting specialisation of the master’s degree, either as a practical class or as the practical component of a seminar. For pharmacy students, PinKo is part of a seminar in the 6th semester offered by the training pharmacy. For medical students, the course was initially offered as an elective in the 7^th^ to 9^th^ semester, supplemented by students in their practical year (PJ). While the first day of the course took place on the FTSK campus in Germersheim with all participating students, the practical training days were carried out at the University Medical Center in Mainz.

The plan was to have approximately 40 interpreting students and about 20 students each from the fields of medicine and pharmacy. After the joint block session, the entire group was divided into two groups in preparation for two practical days per group with identical schedules. The students were distributed in proportion to the number of participants from their departments.

#### 3.2. Learning objectives

The learning objectives were conceived as an extension of the disciplines’ specific learning objective catalogues on conducting conversations. Their aim was to integrate and expand on experiences with and the distinctive features of triadic conversation. Consequently, the first step was to agree on the following superordinate learning objectives:

- developing mutual understanding in the context of the triadic collaboration between the professions as well as a shared understanding of patients’ needs,
- strengthening of the collegial attitude that is necessary for interprofessional cooperation,
- increasing awareness of the intercultural aspects of conducting conversations with patients who do not speak German, and
- expanding general communication and conversation competences.

The sequence of superordinate learning objectives is then reflected in the structure of the developed course. During the joint block session, intra- and interprofessional working groups explore culturally relevant issues, reflecting on and discussing their own perceptions of the patient group and the other professions participating in the discussion. The aim is to develop a shared view of the cultural background of patients who do not speak German, as well as a shared approach to the objectives of the intercultural patient interview. Practicing interpreted consultations and treatment interviews on the practical training day marks the change from primarily cognitive and affective learning objectives to the culturally sensitive expansion of existing conversation skills.

#### 3.3. Course outline 

During the project period from October 2015 to September 2017, the PinKo course was implemented in the 2016 summer semester and the following 2016/2017 winter semester. Table 1 (Tab. 1) shows the schedule, which includes a joint day for all students and the two back-to-back practical training days. 

On the joint preparation day, students of all disciplines take part in impulse lectures, intra- and interprofessional work in small groups, presentations of results and plenary discussions. Two informed-consent interviews or consultations (on diabetes and acute coronary syndrome) previously defined in the project group serve as a common basis for the conversations, both in the medical and pharmaceutical sense. Both cases are prepared as case vignettes that students of all three subjects use to prepare for the interviews. In addition to this, the community interpreting students receive instructions regarding the culture-specific patient roles. Besides personal information, these instructions also include details regarding the patient’s family background, education, occupation, and leisure activities, as well as information regarding their health status and expectations for the interview. Community interpreting students are tasked with enriching the patient role with culturally appropriate behaviours. On the practical training days, preliminary meetings as well as triadic informed-consent interviews and consultations are practised in small groups. The number of language-specific small groups depends on the availability of community interpreting students and teachers from different language groups (Turkish, Arabic, Greek, Chinese, Russian, Polish, Italian and Spanish). As participant observers, the teachers ensure that there is enough time for reflection in the small groups in addition to the practical exercises and that the learning objectives are thus achieved in the best possible way. Where necessary, the teachers guide the feedback process and contribute their professional expertise as appropriate. After the rehearsal phases in the small groups, “interpreting scenarios” of selected language groups take place in the plenary. These conversations are carried out according to the fishbowl principle, meaning that the consultation or informed-consent interview is performed without interruption, surrounded by the participating observers. The performers receive detailed feedback afterwards, both from the observing students and from the teachers of the different professions [39], [40], [41].

#### 3.4. Method

The course was evaluated using a written survey.

In view of the subject matter, the request for socio-demographic information was supplemented with questions regarding previous academic experience in conducting conversations, its applicability in interprofessional and intercultural contexts as well as an assessment of its relevance in the workplace. For the future trained community interpreters, this also includes observations on the respective work cultures in medicine and pharmacy.

In order to assess the development of competences in these areas, a before/after survey was conducted. The focus was on specific questions regarding intercultural knowledge acquisition, the attainment of profession-specific learning objectives and their implementation in joint action in triadic conversation. 

Satisfaction with the course was also assessed, in particular regarding interprofessional teaching and the opportunities to practice in small groups, along with the associated attitudes and approaches regarding collaboration. Due to the different perspectives of the professions and the resulting learning objectives, separate questionnaires were created for each discipline despite the many commonalities.

## 4. Results

A total of 112 students from the participating faculties (Medicine (M) N=8, Pharmacy (P) N=60; Translation (T) N=44) were trained and surveyed regarding the course. The response rate to the survey was almost 90%.

Overall, the event was rated as good (1=very good, 6=insufficient) ((M) 1.67/2.00; (P) 2.29/3.33; (T) 1.50/1.86). A striking feature of this was the lower rating of 2.93 by pharmacy students in the 2016/2017 winter semester (see table 2 (Tab. 2) and attachment 1 ), which was also evident in a comparison with other sub-surveys. The main factors here were the timing of the PinKo course, which the participants perceived as unfavourable, as well as the large number of participants (N=46), which had a limiting effect on the opportunities for practice in relation to the available language groups. The medical students also wished to already start with exercises in the block session and to make the course more compact overall.

PinKo’s contribution to the development of interprofessional competence was corroborated by all students in the post-survey. Thus, for certain challenges in conducting interprofessional conversations (see table 3 (Tab. 3) and attachment 1 ), average competence ratings were good ((M) 1.33-2.00, (P) 2.00-2.93; (T) 1.82-2.25). Compared to the pre-survey, there were differences of 3 or more points on a seven-point Likert scale (1=agree completely, 7=disagree completely) in the assessment of competence before the course.

In the area of the development of intercultural competences, it became clear that the community interpreting students were most likely to benefit in terms of their assessment of their learning progress (see table 4 (Tab. 4) and attachment 1 ). It should be noted here that the awareness of the prospective community interpreters cannot be compared with that of the prospective doctors and pharmacists due to their focus on cultural sensitivity as a basic competence in their field of study. While encounters with foreign-language patients are part of the everyday experience in clinical semesters, the survey shows that there had been no previous courses to increase cultural awareness or opportunities to gain practical experience in interacting with interpreters.

## 5. Discussion

The results of the “Die Triade” project have shown that the PinKo course contributes to the development of interprofessional and intercultural competences. For example, the survey of students from all participating departments confirmed key findings of IPE studies [10], [11], [12], [13], [14], [15], [16], [17]. In addition to improvements in the area of interprofessional communication and collaboration between students of medicine, pharmacy and community interpreting, there was also a significant increase in learning in the area of conducting triadic conversations in the presence of patients. For example, in the context of informed-consent interviews, participants had a clearer understanding of the different roles at the end of the course and were much better able to manage communicative difficulties cooperatively, deliver bad news, address critical aspects or obtain consent for a treatment plan. In this way, involvement of interpreters and the cooperation between healthcare professions contributes significantly to reducing sources of uncertainty for all those involved in the situation [42] and thus makes a valuable contribution to improving treatment and patient safety.

This also applies to (inter-)cultural interaction, which, as expanded agency, assists in better coping with the professional demands of interacting with patients [42]. In this area, the students confirm the professional necessity of dealing with cultural circumstances as a prerequisite for successful action and confirm remarkable learning progress in regard to the intended increase in cultural awareness.

Despite the pilot status and the funding of the project to develop the PinKo course, there were also problems and limitations comparable to the barriers to interprofessional education (IPE) that are described above [11], [14], [15], [19]. For instance, it became apparent that, with a view to sustainability, these issues can only be resolved through the commitment of the project participants to a limited extent. The inclusion of both campus locations for the implementation was not only a challenge in terms of the travel costs that had to be financed, but also in a lack of institutional embedding of interprofessional teaching and the didactic concepts in the respective departments. This also has an impact on the expectations, attitudes and learning behaviour of students and, with a view to student-centred teaching, should be given greater consideration in interprofessional courses. Students of medicine and pharmacy, for example, are clearly more critical than community interpreting students when it comes to weighing the gain in interprofessional and intercultural competences against the amount of travel required for this and making a cross-disciplinary compromise in the presentation of content and the use of alternative methods in the course.

With these aspects in mind, the course is currently being refined, especially since the learning objectives in dealing with difficult conversational situations, including the involvement of an interpreter, will be expanded with the introduction of national learning objectives catalogues in medicine (NKLM) [http://www.nklm.de] and pharmacy (KLP-P) [42]. To this end, initial steps have already been taken to offer learning content, along with training films that have been developed, as a blended learning concept in interactive learning environments. The resulting shorter in-person times also pave the way to integrating other healthcare professions into this interprofessional training format.

## Acknowledgement

We thank the translator Elspeth Noelani Kaiko Lenhard. 

## Profiles

**Name of location: **University Medical Center of Johannes Gutenberg University Mainz (JGU)

**Field of study/Profession: **Medicine

**Number of students per year or semester: **Ca. 140-220 students per semester

**Has a longitudinal communication curriculum been implemented? **Yes

**In which semesters are communicative and social competencies taught? **Semesters 1, 2, 4, 5, 6, 7, 8, 9, 10 

**Which teaching formats are used? **Seminar, course, practical course, lecture, e-learning

**In which semesters are communicative and social competencies tested (formative assessment or relevant for passing and/or graded)? **Semesters 1, 2, 4, 5, 7, 8, 9, 10 

**Which examination formats are used? **Essay, key feature problem examination, poster production, oral-practical, OSCE, patient care report, open book exam, multiple-choice exams

**Who (e.g. clinic, institution) is responsible for the development and implementation? **Decentralised: each institution on its own

## Current professional roles of the authors

- Kai-Uwe R. Strelow, graduate psychologist and economist, staff member of Rudolf Frey Lernklinik (RFLK) at the University Medical Center of Johannes Gutenberg University Mainz (JGU), teaching advisor, division head Human Factors, Interpersonal Competencies, Treatment and Patient Safety
- Dr. phil. Şebnem Bahadır, graduate translator/interpreter, senior lecturer and module coordinator for “Specialised interpreting in healthcare, social and public services”, Department of Intercultural German Studies, Faculty of Translation, Language and Cultural Studies, Johannes Gutenberg University Mainz (JGU), since December 2020 Professor of Translation Studies, Research Focus on Translation, Migration and Minorities, ITAT, University of Graz.
- Dr. rer. nat. Bettina Stollhof, head pharmacist at Kreuznacher Diakonie in Bad Kreuznach (Germany), specialist pharmacist for clinical pharmacy and theoretical and practical training, lecturer in clinical pharmacy at Johannes Gutenberg University Mainz (JGU)
- Dr. rer. nat. Rita Marina Heeb, specialist pharmacist for clinical pharmacy, head of quality control, deputy QP and hygiene officer at the pharmacy of the University Medical Center of Johannes Gutenberg University Mainz (JGU), lecturer in pharmacoepidemiology and pharmacoeconomics
- Dr. med. Holger Buggenhagen MME, Head of Rudolf Frey Lernklinik (RFLK) at the University Medical Center of Johannes Gutenberg University Mainz (JGU), anaesthesiologist with additional qualifications in emergency-care medicine and specialised anaesthesiological intensive-care medicine

## Competing interests

The authors declare that they have no competing interests. 

## Supplementary Material

Table A1: variables used

## Figures and Tables

**Table 1:**
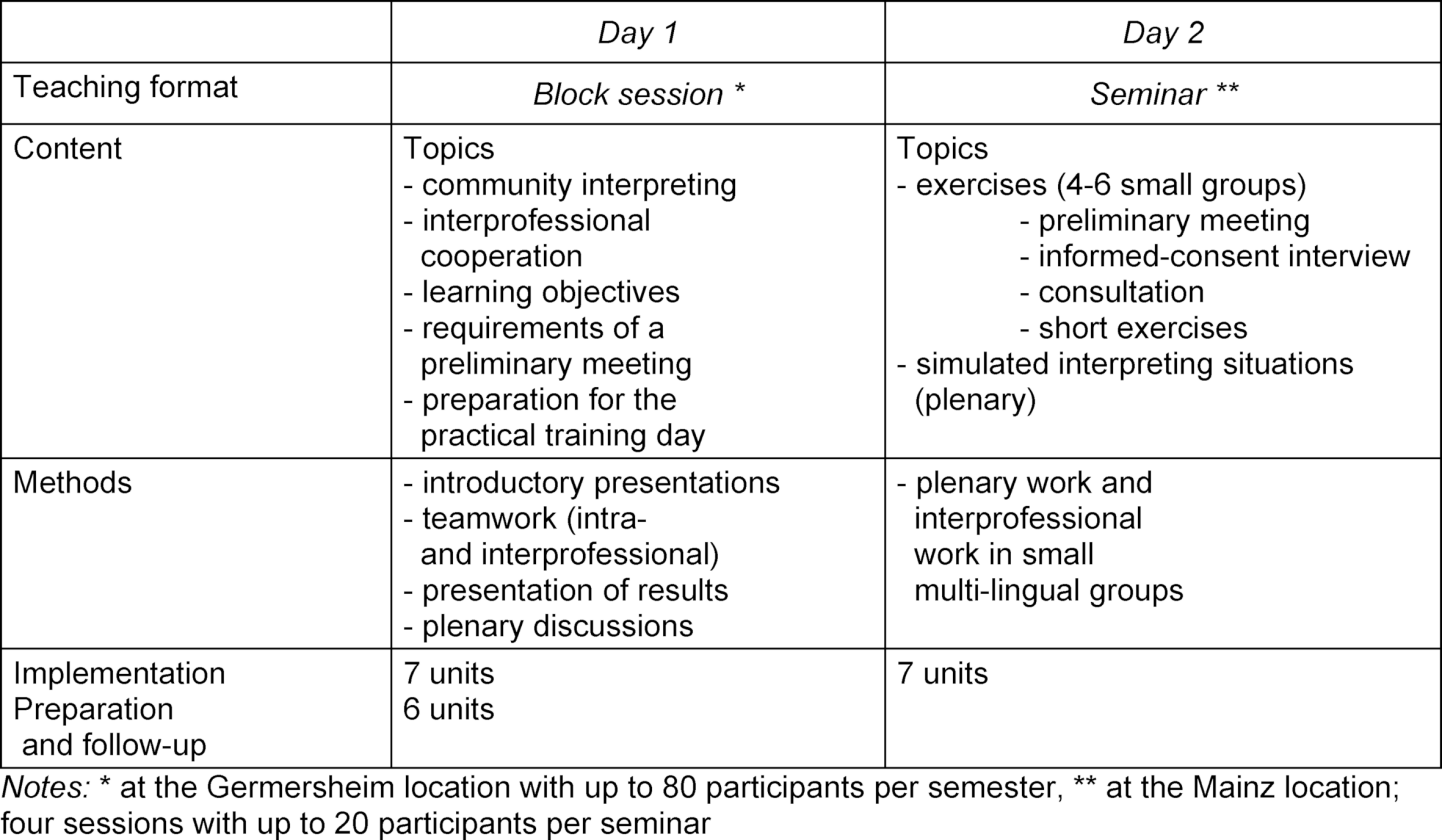
PinKo course overview

**Table 2:**
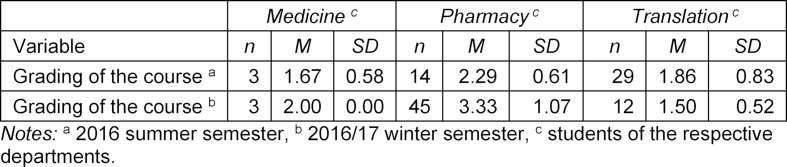
Descriptive statistics of the variables for the overall evaluation of the event

**Table 3:**
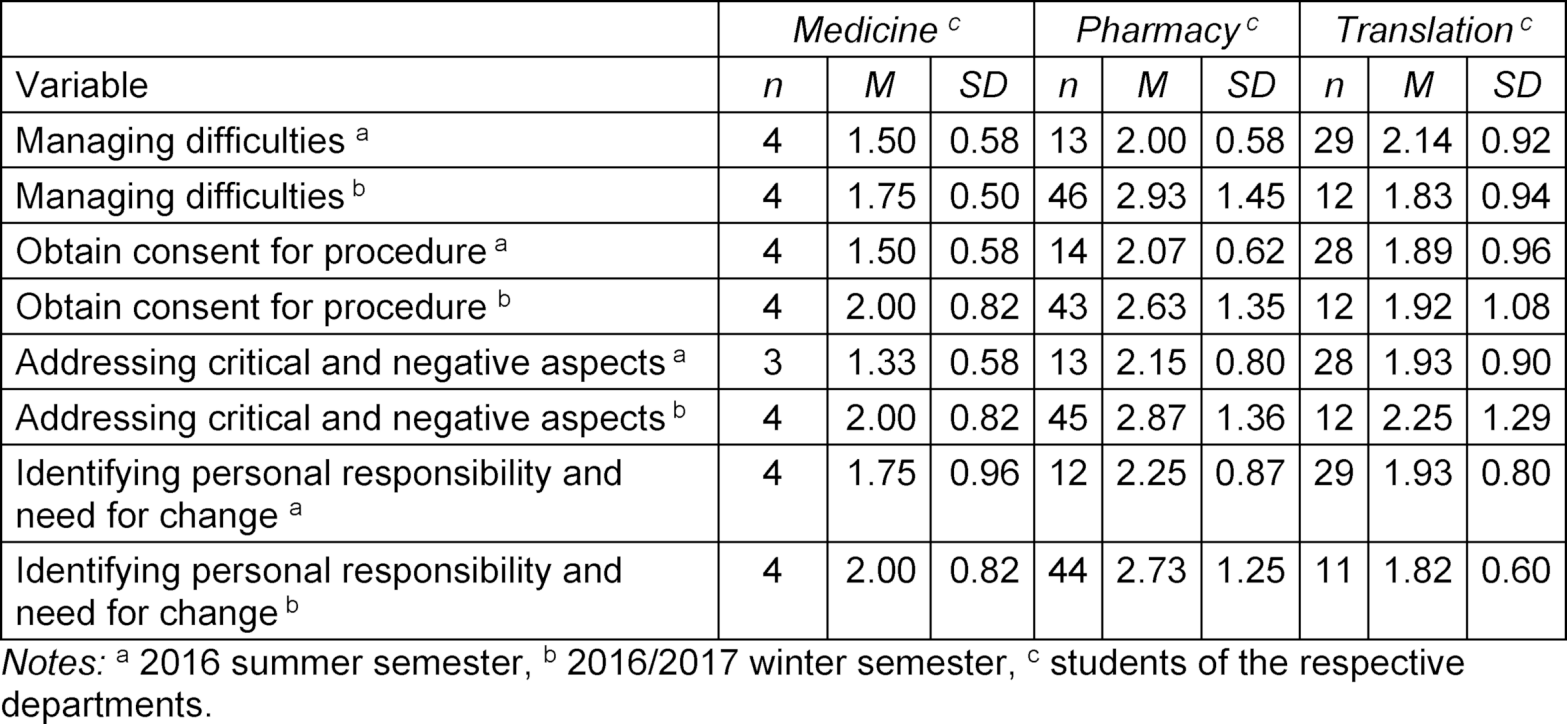
Descriptive statistics of the variables on learning progress in the area of interprofessional work

**Table 4:**
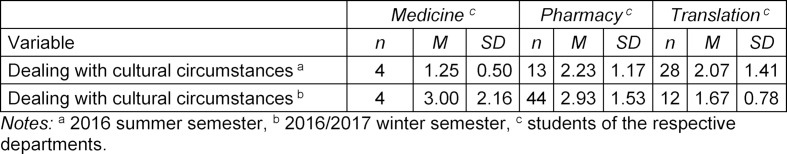
Descriptive statistics for the variables on learning progress in the area of intercultural competence
