# Supplementary material for: Patient interviews in interprofessional and intercultural contexts (PinKo) – project report on interdisciplinary competence development in students of medicine, pharmacy, and community interpreting
Source: GMS J Med Educ. 2021 Mar 15;38(3):Doc67. doi: 10.3205/zma001463 (PMC7994884; doi:10.3205/zma001463)
Supplement: Table A1: variables used [file JME-38-3-67-s-001.pdf]

Attachment 1: Table A1: *Variables used*

Variable name	Description	Response
Overall grade for the course	If you had to give the entire course a grade (from 1= “very good” to 6= “insufficient”), what grade would you award?	1 = very good 2 = good 3 = satisfactory 4 = sufficient 5 = poor 6 = insufficient
Repeat participation	Would you take part the course again?	0 ...100 (in percent)
Dealing with cultural circumstances	During the course, I learned to consider cultural and social circumstances.	1= agree completely to 7= disagree completely
Manage difficulties	During the course, I learned how to manage difficulties in communicating/cooperating with the interpreter (e.g. during the patient interview).	1= agree completely to 7= disagree completely
Obtain approval for the procedure	During the course, I learned how to obtain the patient’s consent for the procedure in cooperation with the interpreter.	1= agree completely to 7= disagree completely
Addressing critical and negative aspects	During the course, I learned how to address negative and critical aspects in cooperation with the interpreter.	1= agree completely to 7= disagree completely
Identify personal responsibility and need for change	During the course, I learned how to work in cooperation with the interpreter to point out the patient’s own responsibility and the need for change.	1= agree completely to 7= disagree completely
